# EBER2 RNA-induced transcriptome changes identify cellular processes likely targeted during Epstein Barr Virus infection

**DOI:** 10.1186/1756-0500-1-100

**Published:** 2008-10-28

**Authors:** Sebastian Eilebrecht, François-Xavier Pellay, Peter Odenwälder, Guillaume Brysbaert, Bernd-Joachim Benecke, Arndt Benecke

**Affiliations:** 1 Department of Biochemistry; Ruhr University Bochum; Universitätsstr, 150; 44780 Bochum; Germany; 2 Institut des Hautes Études Scientifiques & Institut de Recherche Interdiciplinaire – CNRS USR3078 – Université Lille1; 35, route de Chartres; 91440 Bures sur Yvette; France

## Abstract

**Background:**

Little is known about the physiological role of the EBER1 and 2 nuclear RNAs during Epstein Barr viral infection. The EBERs are transcribed by cellular RNA Polymerase III and their strong expression results in 10^6 ^to 10^7 ^copies per EBV infected cell, making them reliable diagnostic markers for the presence of EBV. Although the functions of most of the proteins targeted by EBER RNAs have been studied, the role of EBERs themselves still remains elusive.

**Findings:**

The cellular transcription response to EBER2 expression using the wild-type and an internal deletion mutant was determined. Significant changes in gene expression patterns were observed. A functional meta-analysis of the regulated genes points to inhibition of stress and immune responses, as well as activation of cellular growth and cytoskeletal reorganization as potential targets for EBER2 RNA. Different functions can be assigned to different parts of the RNA.

**Conclusion:**

These results provide new avenues to the understanding of EBER2 and EBV biology, and set the grounds for a more in depth functional analysis of EBER2 using transcriptome activity measurements.

## Findings

### Background

Epstein-Barr virus (EBV) is a member of the herpesvirus family present in almost the entire human adult population [1-3] and has been found to be associated with oncogenesis of Burkitt's lymphoma, B- and T-cell leukemia/lymphomas, nasopharyngeal carcinoma, breast cancer and gastric cancer [4-7]. In EBV infected cells, several viral genes are expressed of which the six nuclear antigens (EBNAs 1–6), the three membrane proteins (LMP-1, -2A and -2B) and the small non translated EBER1 and EBER2 RNAs are the most abundant [1,3].

The EBERs are transcribed by cellular RNA Polymerase III (polIII) and their strong expression results in 10^6 ^to 10^7 ^copies per EBV infected cell [8,9], making them reliable diagnostic markers for the presence of EBV. EBERs are located in the nucleus [10] and have been shown to bind to several cellular proteins, such as the La antigen [9,11-14], the EBER-associated protein (EAP, now referred to as ribosomal protein L22) [15,16], and the interferon-inducible protein kinase R (PKR) [17,18]. The binding of EBER1 to PKR blocks the PKR pathway resulting in the resistance of the cell to Fas-mediated apoptosis [19]. Although the functions of most of the proteins targeted by EBER RNAs have been studied, the role of EBERs themselves still remains elusive.

EBER-induced interleukin (IL)-10 expression in Burkitt's lymphoma (BL) cells has been demonstrated [20]. IL-10 is suggested to be an autocrine growth factor for BL cells [21], and hence potentially links EBER expression to hyperplastic transformation. Furthermore, a recent report indicates that rather EBER2 than EBER1 plays a central role in B-cell growth transformation [22]. Given this indication of a transcriptional response to EBER expression, as well as the indication of the functional implication of EBER2 in cellular transformation, we used microarrays to assess transcriptome changes following expression of EBER2 in HEK 293 cells.

## Results and discussion

**Figure 1:**
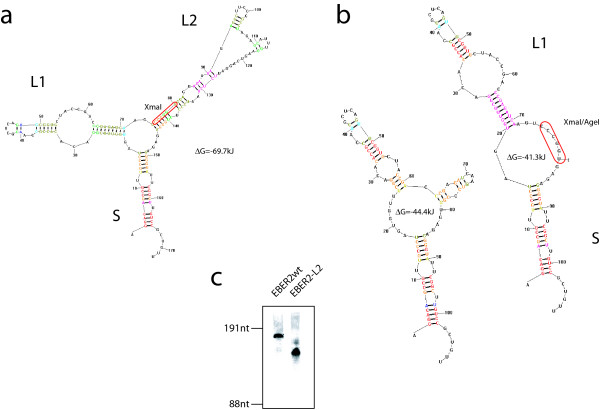
**a. Predicted secondary structure of the wild-type EBER2 RNA.** The 5'3' stem ("S"), and the two loops ("L1", "L2"), as well as the XmaI cloning site used to construct the EBER2-L2 mutant are indicated. b. Alternate predicted secondary structures for the EBER2-L2 RNA are illustrated. Labeling as in (a). Both structures differ minimally in their free energy (ΔG = 3.1 kJ). c. Northern blot analysis of transfected HEK293 cells confirming the expression of both EBER2 constructs at comparable levels.

To obtain an EBER2 wild-type expression plasmid the complete gene sequence including all polIII expression elements from -156 to +195 was cloned into pUC18. Since EBER backbones have been proposed as vehicles for expression of short hairpin RNAs [23], we constructed an EBER2 expression plasmid lacking the entire loop 2 (EBER2-L2) by introducing an AgeI restriction site at position +142 which was then restricted and religated with the naturally occurring XmaI site at position +77 (Figure 1). The secondary structures of EBER2/EBER2-L2 were calculated [24] and illustrated in Figure 1. The two alternate EBER2-L2 structures differ in their free energy by 3.1 kJ, and retain the entire 5'3' stem structure (S) as well as the entire or the upper part of the loop 1 (L1). Both constructs express similar amounts of RNA as confirmed by northern blotting using a specific ^32^P-labelled antisense RNA probe that cross-hybridizes with both (Figure 1C).

**Figure 2:**
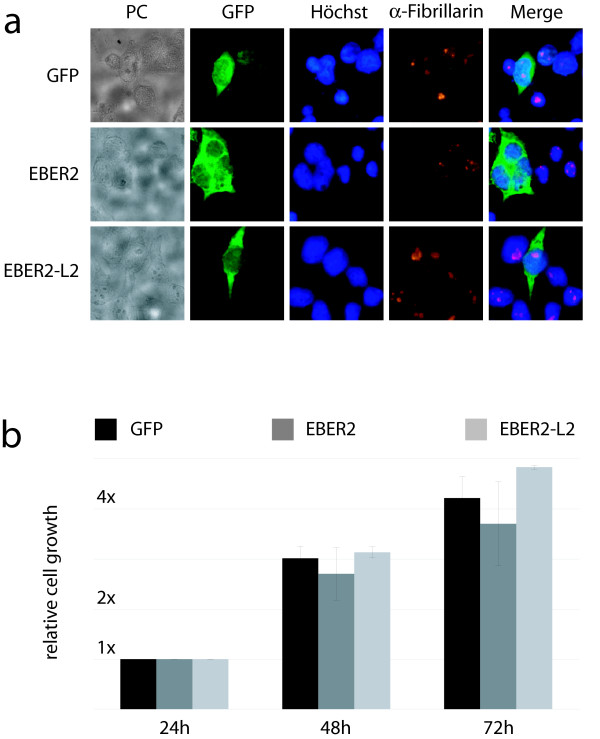
**Absence of effects on cellular growth and subcellular organization by EBER2 expression.** a. Phase-contrast ("PC"), fluorescence ("GFP", "Hoechst"), and immunofluorescence ("α-Fibrillarin") for mock ("GFP") and EBER2 or EBER2-L2 transfected HEK293 cells. b. Growth curves for the same cells. Averages and standard deviations of at least three independent transfections are shown.

First, we verified the absence of effects on cellular growth or organization during ectopic expression of the RNAs. Vectors that co-express EBER2/EBER2-L2 RNAs and green fluorescent protein (GFP) were generated by inserting the EBER2 constructs into the EcoO109I restriction site of peGFP-N1 vector (Clontech). HEK293 cells cultured in DMEM (Sigma) supplemented with 10% (v/v) Fetal Bovine Serum (Bio West) at 7% CO_2 _and 37°C were transfected using FUGENE-HD (Roche), and GFP positive cells (~80%) were counted daily. Nuclei were stained with Hoechst 33342 (Invitrogen), and Anti-Fibrillarin antibody (ab5821, Abcam) was used as a marker for nucleoli by immunostaining of Fluoromount-G mounted cells. Neither analysis revealed significant differences when comparing EBER2, EBER2-L2 and mock (GFP only) transfected cells (Figure 2). We also were not able to detect any other significant changes in the morphology of the HEK293 cells upon EBER2 expression.

For microarray analysis, HEK293 cells were transfected with pUC18 vector or the EBER2/EBER2-L2 constructs by calcium phosphate co-precipitation technique using the initial constructs to avoid secondary effects by the co-expression of GFP. Total RNA was extracted 48 h post-transfection with an RNeasy Midi Kit (Qiagen), and analyzed for quality with an Agilent 2100 Bioanalyser. RNA amplification, labeling, hybridization and detection were done following the protocols supplied by Applied Biosystems using 2 μg of total RNA as input. Labeled cRNAs were then hybridized and detected on HGS Version 2.0 AB1700 microarrays containing probes for 29362 validated human genes. The AB1700 technology has been demonstrated to cover an increased signal dynamic range, display higher sensitivity and provide more robust gene expression estimates when compared to the leading competing technologies [25].

**Figure 3:**
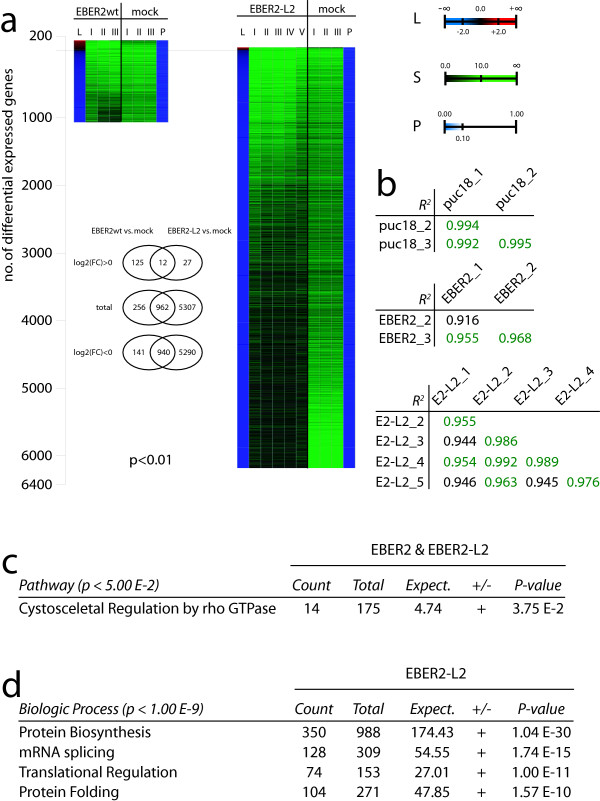
**a. Heat-maps of statistically significant (p < 0.01) changes in gene expression when comparing EBER2/EBER2-L2 transfected to mock (pUC18) transfected cells at 48 h post-transfection. **"L": log2(FoldChange), roman numbers indicate independent biologic replicates, "S": signal; "P": p-value. b. Average Pearson correlation coefficients of the transcriptome profiles of the biological replicates. Homogeneity and reproducibility of the independent biologic replicates was assessed using Pearson correlation coefficients averaged over the common subset of ~34000 probes. Pearson correlation coefficients larger than 0.950 have been highlighted in dark green. c. Pathway analysis of the joint set of EBER2 and EBER2-L2 target genes. "Count": number of genes corresponding to a given pathway present in the selected dataset, "Total": total number of genes associated to given pathway, "Expect.": expected number of genes given the size of the selected dataset and a random zero-hypothesis, "+:-": statistical over- (+) or under- (-) representation, "P-value": likelihood of zero-hypothesis to be correct. d. Biologic Process analysis of EBER2-L2 target genes. Nomenclature as in (b).

In this manner three independent biologic replicates (independent transfections) for the pUC18 and EBER2 plasmids, and five independent biologic replicates for the EBER2-L2 construct were generated. Expression Array System Software V1.1.1 (ProdNo: 4364137) was used to acquire the images and primary data analysis. The homogeneity of the different biologic replicates was assessed using heat-maps (Figure 3A) and average Pearson correlation coefficients calculated over the entire set of ~34000 probes (Figure 3B). All three sets of experiments were found to be highly reproducible as demonstrated by *R*^2^s of at least 0.92, with 75% of the *R*^2^s being even superior to 0.95 (Figure 3B). Calculation of subtraction profiles was performed according to standard procedures in an "everyone-against-everyone" scheme and log2 fold-changes ("logQ", "L") where then determined as averages of variance-weighted individual logQ values using the NeONORM method for normalization (Additional File 1; M4) with parameter *k *= 0.20. P-values were determined using standard ANOVA methodology. Multiple probes for a single gene, cross-reactivity of a single probe to several genes, as well as the resolution of probe-ID annotations were according to previously defined standards (Additional File 1; M5).

Statistically significant changes in the HEK293 transcriptome upon EBER2 or EBER2-L2 expression are observed (Figure 3A). In the former case a total of 1218 probes are found to detect differential gene expression at p < 0.01, of which 137 are stimulated, and 1081 are repressed in their expression when compared to mock (pUC18) transfected cells. Thus EBER2 expression has a significant impact on the cellular transcriptome. Likewise, the EBER2-L2 has a very pronounced effect on cellular transcription. A total of 6269 probes are found to return statistically significant (p < 0.01) differential signals, with the vast majority of genes being repressed in their expression (6230 probes). Comparisons of both target gene sets (Additional Files 2, 3) reveal that out of the 1218 EBER2 targets 962 (or 79%) are also found to be EBER2-L2 targets (Venn-Diagrams, Figure 3A; Additional File 4). The clear majority of genes specific to EBER2 are up-regulated in their expression indicating that positive transcription regulation requires integrity of, and may be dependent directly on the L2 structure. The important number of genes negatively affected in their transcription by EBER2-L2 is believed to be a consequence of functionally unproductive titration of cellular components leading to global repression of RNA processing in the cell. Alternatively, albeit very unlikely, we can not formally rule out the possibility that EBER2 RNA or degradation products of the RNA interfere with the transcriptome activity measurements. Such findings have to be considered when using EBER2 RNA sequences as a vehicle for the expression of shRNAs, as recently proposed by Choy and colleagues [23].

**Figure 4:**
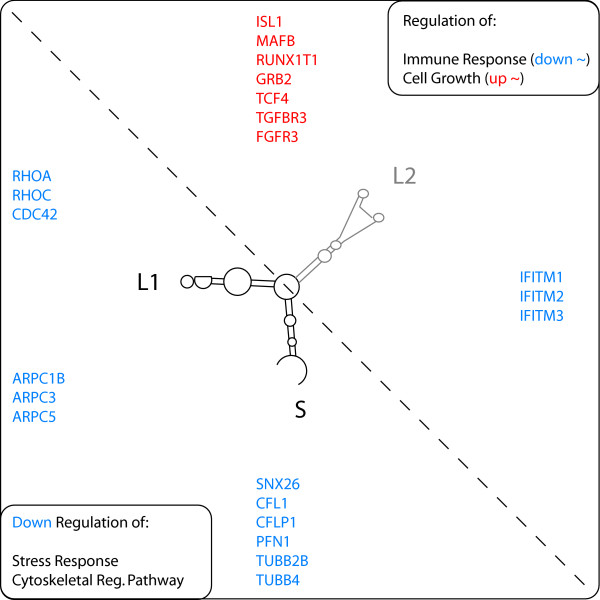
**Schematic representation of the main results.** EBER2 down-regulated genes in blue, up-regulated target genes in red. The L2 is required for positive regulation of cell growth related genes and negative regulation of interferon target genes. The core (S+L1) of the EBER2 RNA targets the "cytoskeletal regulation by rho GTPases" pathway.

Functional meta-analysis using Kegg/GO/PANTHER ontologies for emergent properties of the two target gene sets was performed using a binominal distribution and a Bonferroni multiple testing correction. The joint EBER2/EBER2-L2 target gene set is statistically significantly (p < 0.05) enriched in genes coding for components of the "cytoskeletal regulation by rho GTPase" pathway (Figure 3C). Both RHOA and RHOC coding genes, as well as CDC42 were found to be repressed across the eight independent biologic replicates when compared to the mock transfected cells, and have previously been shown to be primordial in the cellular stress response pathway [26] by orchestrating the regulation of cytoskeletal dynamics [27]. Concomitantly, we find other cellular components of the cytoskeleton among the EBER2 target genes, such as the actin related protein 2/3 complex family members 1B, 3, and 5 [28], as well as the tubulin beta 2B and 4 family members [29] along with other cytoskeletal components. These data strongly suggest negative regulatory responses of the rho-pathway following EBER2 expression (Figure 4; Additional File 5).

The strong negative regulation by EBER2-L2 is explained by over-representation (p < 1.00 E-9) of genes related to mRNA maturation and protein synthesis in this dataset (Figure 3D). EBER2-L2 likely interferes in a non-physiological manner with the production and/or assembly of the splicing and translation apparatuses, as the same processes are not overrepresented when the EBER2 target gene set is analyzed. Furthermore, both EBER2 and EBER2-L2 might be processed by the RNA editing/degradation machinery and give rise to molecules inferring with either physiological processes, or, albeit highly unlikely, the transcriptome analysis itself.

The EBER2 specific target gene repertoire also reveals down-regulation of three highly related interferon inducible trans-membrane proteins IFITM1, 2, 3, and induction of several cellular growth regulators such as FGF receptor 3 and TGFβ receptor 3 [30]. The regulation of the latter is observed only with EBER2 and therefore is directly assignable to L2 integrity (Figure 4). The main categories of EBER2 targeted genes related to cellular growth, reorganization, and stress thereby seem to coincide well with previous advances in the understanding of EBER2 biology [22]. The results here should therefore provide a novel, complementary avenue to the understanding of EBER RNAs and EBV physiopathology.

## Conclusion

These investigations establish the feasibility of recording transcriptome dynamics downstream of non-translated small nuclear RNAs. Furthermore, they do not only point to a role of EBER2 in regulating the activity of cellular pathways related to growth and the cytoskeleton, as well as negative interference into interferon mediated immune responses and cellular stress, but also provide a basis for the identification of the molecular targets of EBER2 RNA and the understanding of the physiopathology of EBV, in general. Regulating the activity of growth, cytoskeletal dynamics as well as inhibition of cellular stress seems in tune with recent advances in the understanding of EBER2 biology in the context of B-cell transformation [22]. Finally, these data should also prove very helpful in the interpretation of results obtained using shRNA-EBER fusion constructs as pioneered by Choy and colleagues [23]. Given its high expression levels and structural stability, EBER2, like other pol-III transcribed and/or viral RNAs are being proposed as vehicles for the over-expression of knock-down small interfering RNA species. The fact that the backbone might, as shown here in the case of the EBER2, have profound effects on the cellular transcriptome will certainly have to be studied during the validation of such hybrid molecules.

## Competing interests

The authors declare that they have no competing interests.

## Authors' contributions

SE has designed and generated the EBER2 expression constructs and performed the molecular and cellular biology experiments, assisted during transcriptome data generation, and contributed to the writing of the manuscript. FXP has performed the transcriptome data acquisition and primary, secondary, and tertiary transcriptome data analysis. PO has contributed to the molecular and cellular biology experiments as well as manuscript preparation. GB has programmed the algorithm used for the ontology meta-analysis. BJB and AB have designed and supervised the study, performed the biological interpretation of the data, and written the manuscript. All authors have read and approved the final manuscript.

## Supplementary Material

Additional File 1 A description of the methodology used, including additional references. Click here for file

Additional File 2 An ascii tab-delimited text file containing the logarithmic base-two fold-change, p-value, and primary annotation for the entire set of probes revealing statistically significant changes in gene expression (p < 0.01). Click here for file

Additional File 3 Idem for EBER2-L2 in above format. Click here for file

Additional File 4 The joint target gene set in above format. Click here for file

Additional File 5 EBER2 targets from the "cytoskeletal regulation by rho GTPases" pathway. Click here for file
