# Supplementary material for: EBER2 RNA-induced transcriptome changes identify cellular processes likely targeted during Epstein Barr Virus infection
Source: BMC Res Notes. 2008 Oct 28;1:100. doi: 10.1186/1756-0500-1-100 (PMC2588618; doi:10.1186/1756-0500-1-100)
Supplement: Additional File 1 — A description of the methodology used, including additional references. [file 1756-0500-1-100-S1.pdf]

Methods – Eilebrecht et al. 2008 – BMC Res. Notes

1.1 Constructs

EBER2 wild type

In order to obtain an EBER2 wild type plasmid the complete gene sequence from -156 to +195 was restricted by Sau3A (NEB) and ligated into the BamHI site of the pUC18 vector (Fermentas) [M1].

EBER2-L2

To eliminate the loop structure from +77 to +142 of the EBER2-RNA, an AgeI restriction site was introduced at +142 of the EBER2 wild type plasmid by site directed mutagenesis of the pUC18-EBER2 plasmid. The resulting intermediate clone was restricted by XmaI and AgeI (NEB) and religated to obtain the EBER2-L2 construct.

Structure prediciton

The secondary structure of the constructs was calculated by RNAstructure Version 4.4 [M2]. The maximal energy difference was set to 10%.

1.2 Cell culture

Cell culture conditions

HEK 293 cells were cultured in NUNC™ cell culture dishes in DMEM (Sigma) supplemented with 10% (v/v) Fetal Bovine Serum (Bio West) at 7% CO_2 and 37°C.

Transfection

One day before transfection $1x10^6$ cells were seeded in a cell culture dish (d=94mm) and incubated under standard conditions. At 50% density the medium was exchanged and the cells were incubated at 3% CO_2 and 37°C for 3 hours. Transfection was performed by calcium phosphate coprecipitation method with 10µg of DNA per dish. Cells were incubated again for 48 hours under standard conditions before being assayed.

1.3 RNA extraction, analysis, labelling and hybridization

RNA extraction

Total RNA from transfected HEK 293 cells was extracted with an RNeasy Midi Kit (Quiagen) as recommended by the manufacturer.

RNA analysis

Total RNA was analysed with an Agilent 2100 Bioanalyser (Agilent Technologies) as recommended by the manufacturer.

RNA labeling, hybridization, detection

RNA amplification, labelling, hybridization and detection were done following the protocols supplied by Applied Biosystems together with the corresponding kits. 2 µg of total RNA were submitted to RT-IVT amplification and labelling (Applied Biosystems, ProdNo: 4339628). Labelled cRNAs were then hybridized and detected according to the supplied protocols (Applied Biosystems, ProdNo: 4336875).

1.4 Microarray

AB1700 Microarray Technology

Transcriptome profiles were acquired using novel Applied Biosystems AB1700 technology. This microarray platform has been demonstrated to cover an increased signal dynamic range, display higher sensitivity and provide more robust gene expression estimates when compared to the leading competing technologies due to different, chemiluminescence-based detection chemistry [M3]. All experimental data referred to as HGS Version 2.0 (Human Genome Survey Version 2, ProdNo: 4359029) used in this manuscript were generated on Applied Biosystems AB1700 transcriptome platform (ProdNo: 4338036). These arrays contain probes for 29362 validated human genes.

1.5 Data analysis

Data pre-processing and primary analysis

Applied Biosystems Expression Array System Software Version 1.1.1 (ProdNo: 4364137) has been used to acquire the chemiluminescence and fluorescence images and primary data analysis. Briefly, the primary analysis consists of the following individual operations:

[1] Image correction: Calibration images are used to subtract any device-dependent bias from the raw images and to correct the spectral bleed-through from the chemiluminescence (CL) into the fluorescence (FL) channel. Pixels that are saturated in the long exposure (25s) CL image are replaced by appropriately scaled values of the short exposure (5s) CL image.

[2] Global and local background correction: global correction for non-specific signals and unwanted hybridization, using specific random-sequence oligonucleotide control spots and local correction for bleeding between adjacent probes with high intensity differences. A single bias is calculated using pixels inside an annulus around the feature aperture.

[3] Feature normalization: Compensation of spotting variations (comparable to print tip normalization) and optical trends. Normalization of chemiluminescence CL intensities by FL - CL ratios: In this step, the coefficient of variance estimate is calculated, too.

[4] Spatial normalization: Spatial trend correction on the feature level using specific (SPN) control spots evenly placed over the array (~1 SPN control in 300 spots). Spatial normalization mainly captures non-uniform illumination of the array.

[5] Global normalization: Division of all signals by the median. Note, that we renormalize the resulting data according to the median once more after having removed probes for which the Applied Biosystems Software has set flags equal or greater 2^{12}, indicating compromised or failed measurements (as recommended by Applied Biosystems).

Data processing and statistical analysis

Calculation of subtraction profiles was performed according to standard procedures with the following modifications: data from different biological conditions were compared in an "everyone against everyone" scheme and log2 quotients ("logQ", "L") were then determined as averages of weighted individual logQ values. For these inter-assay comparisons the NeONORM method was used for normalization using k=0.20 [M4]. P-values were determined based on a normal distribution hypothesis of signal intensities using standard ANOVA methodology. Multiple probes for a single gene, cross-reactivity of a single probe to several genes, as well as the resolution of probe-ID annotations was done according to the standards defined previously [M5]. Heat-maps were created according to standard methods. In order to underline the single-colour chemiluminescence detection character of the AB1700 system we use a blank (signal=0) to bright green (signal>10) gradient to represent signal intensities.

1.6 Pathways

Combining GO, Kegg and PANTHER annotations, we assigned all probes present on the HGS Version 2.0 array to the PANTHER pathways. We then calculated the relative representation of those probes detected as significantly regulated. P-values for over- and under-representation of pathways were calculated using a binominal distribution.

References

[M1] Baer, R., A. T. Bankier, M. D. Biggin, P. L. Deininger, P. J. Farrell, T. J. Gibson, G. Hatfull, G. S. Hudson, S. C. Satchwell, C. Seguin, P.S. Tuffnell, and B.G. Barrell: **DNA sequence and expression of the B95-8 Epstein-Barr virus genome**. *Nature* 1984, **310:**207-11.

[M2] Mathews, D. H., M. D. Disney, J. L. Childs, S. J. Schroeder, M. Zuker, and D. H. Turner: **Incorporating chemical modification constraints into a dynamic programming algorithm for prediction of RNA secondary structure**. *Proc Natl Acad Sci U S A* 2004, **101:**7287-92.

[M3] Noth, S., G. Brysbaert, F. X. Pellay, and A. Benecke: **High-sensitivity transcriptome data structure and implications for analysis and biologic interpretation**. *Genomics Proteomics Bioinformatics* 2006, **4:**212-29.

[M4] Noth, S., G. Brysbaert, and A. Benecke: **Normalization using weighted negative second order exponential error functions (NeONORM) provides robustness against asymmetries in comparative transcriptome profiles and avoids false calls**. *Genomics Proteomics Bioinformatics* 2006, **4:**90-109.

[M5] Noth, S., and A. Benecke: **Avoiding inconsistencies over time and tracking difficulties in Applied Biosystems AB1700/Panther probe-to-gene annotations**. *BMC Bioinformatics* 2005, **6:**307.
